# Flux-P: Automating Metabolic Flux Analysis

**DOI:** 10.3390/metabo2040872

**Published:** 2012-11-12

**Authors:** Birgitta E. Ebert, Anna-Lena Lamprecht, Bernhard Steffen, Lars M. Blank

**Affiliations:** 1 Institute of Applied Microbiology (iAMB), RWTH Aachen University, Worringer Weg 1 52074 Aachen, Germany; Email: birgitta.ebert@rwth-aachen.de (B.E.E.); 2 Service and Software Engineering, University of Potsdam, August-Bebel-Straße 89, 14482 Potsdam, Germany; Email: lamprecht@cs.uni-potsdam.de (A.L.L.); 3 Programming Systems, TU Dortmund University, Otto-Hahn-Str. 14, 44227 Dortmund, Germany; Email: bernhard.steffen@cs.tu-dortmund.de (B.S.)

**Keywords:** ^13^C metabolic flux analysis, MFA, high-throughput analysis, scientific workflows, workflow management, Bio-jETI

## Abstract

Quantitative knowledge of intracellular fluxes in metabolic networks is invaluable for inferring metabolic system behavior and the design principles of biological systems. However, intracellular reaction rates can not often be calculated directly but have to be estimated; for instance, via ^13^C-based metabolic flux analysis, a model-based interpretation of stable carbon isotope patterns in intermediates of metabolism. Existing software such as FiatFlux, OpenFLUX or 13CFLUX supports experts in this complex analysis, but requires several steps that have to be carried out manually, hence restricting the use of this software for data interpretation to a rather small number of experiments. In this paper, we present Flux-P as an approach to automate and standardize ^13^C-based metabolic flux analysis, using the Bio-jETI workflow framework. Exemplarily based on the FiatFlux software, it demonstrates how services can be created that carry out the different analysis steps autonomously and how these can subsequently be assembled into software workflows that perform automated, high-throughput intracellular flux analysis of high quality and reproducibility. Besides significant acceleration and standardization of the data analysis, the agile workflow-based realization supports flexible changes of the analysis workflows on the user level, making it easy to perform custom analyses.

## 1. Introduction

Metabolic flux distributions represent the integrated network responses of the different cell components (genes, mRNAs, proteins, and metabolites) and abiotic physico-chemical system parameters. The quantitative knowledge of these fluxes is of high importance in deciphering cellular functions and guiding rational strain engineering for industrial biotechnology. ^13^C metabolic flux analysis is currently the most sophisticated and reliable method for determining intracellular reaction rates and has become a widely used tool in systems bio(techno)logy.

Because the demand for quantitative metabolic flux data is increasing, the quality and quantity of analytical results increases, too. Especially new protocols for cell cultivation, sample handling, and sample analysis by mass spectroscopy are driving these developments [1]. While early publications rarely presented more than six flux distributions, the first examples exist that include 30 [2] or even more than 150 data sets [3,4]. Currently, available software tools for ^13^C-based flux analysis, such as FiatFlux [5], OpenFlux [6], 13CFLUX [7] and the updated version 13CFLUX2 [8] require (intensive) user interactions and expert knowledge, as GC-MS data quality and relevance have to be assessed. Yet, these interactive data evaluation workflows can become limiting when hundreds of data sets have to be handled. Ideally, automated software versions would be available that calculate high quality flux distributions in the metabolic network under study using labeling and physiological data with a minimal need of user interaction. Consequently, in this study we aimed to translate the user interactions and expert knowledge required for the analysis into quantifiable criteria suited for the automated determination of intracellular flux distributions. 

### 1.1. Metabolic Flux Analysis

Metabolic flux analysis (MFA) is applicable for systems that are in a pseudo-steady state. Under this condition, the differential equation system of metabolite mass balances reduces to a linear equation system, which relies solely on the known stoichiometry of the biochemical reaction network. However, the system is often underdetermined if only constrained by extracellular uptake and secretion rates and the growth rate of the cell, with the consequence that not all fluxes, especially those of parallel pathways and cyclic fluxes in the network, can be resolved. Additional constraints can be gained from growth experiments, in which cellular growth substrates labeled with stable isotope tracers like ^13^C are fed to the biological system [9]. The labeled (carbon) atoms are then distributed over the metabolic network by incorporation into intracellular metabolites and conserved in amino acids located in proteins, whose labeling patterns can be measured by nuclear magnetic resonance (NMR) [10] or mass spectrometry (MS) instruments [11]. The rationale behind these ^13^C tracer experiments is that the carbon backbones of the metabolites are often manipulated differently by alternative pathways, resulting in distinct ^13^C labeling patterns of the metabolites. Thus, constraints on fluxes complementary to the basic stoichiometric constraints can be derived by measuring the mass isotope distribution of metabolites, that is, the relative abundances of molecules only differing in the number of heavy isotopes. Ideally, these additional constraints render the system fully resolvable.

Two main approaches exist for the interpretation of the determined ^13^C data and the calculation of intracellular flux distributions: Global isotopomer balancing [6,12,13,14] and metabolic flux ratio analysis [10,15]. Both approaches have their advantages and disadvantages as follows: In the global isotopomer ((mass) isotope isomer) balancing approach, metabolic fluxes are estimated from the isotopomer measurements by iteratively generating candidate flux distributions until they fit well to the experimental ^13^C labeling [6,12,13,14]. The challenge of this nonlinear optimization problem is to find the global optimum, which make this approach demanding in computation (time) and requires data of equally high accuracy as often all data are used unweighted. Existing software applying this approach include ^13^C-FLUX(2) [7,8] and OpenFLUX [6]. ^13^C-FLUX is a comprehensive tool enabling the analysis of different models and isotopic tracers. Besides the calculation of the flux distribution it offers a statistical analysis of the determined fluxes. Drawbacks of the software are its restriction to Linux or Unix OS, the requirement of the user to specify free fluxes and initial guesses of the flux distribution, the manual initiation and termination of simulation runs and the demanding computation power and time. The updated version ^13^C-FLUX2 features several improvements such as reduced computation times, improved data exchange and flux distribution visualization. OpenFLUX, a completely open source MATLAB-based software, features facilitated model generation and short computation times applying the Elementary Metabolite Unit algorithm. [15], also implemented in ^13^C-FLUX2. For both software packages ^13^C data have to be preprocessed externally.

Metabolic flux ratio analysis, coined by Sauer as METAFoR [15], relies on the local interpretation of labeling data using probabilistic equations that constrain the ratios of fluxes producing the same metabolite. The approach is mainly independent of the global flux distribution in the entire metabolic network [15,17,18], with the consequence that flux ratios can be calculated without knowing the uptake and production rates of external metabolites and the biomass composition of the cell. If enough independent flux ratios can be identified, it is possible to use them to constrain the metabolic network equation system and to calculate the full flux distribution of the network [19]. In contrast to the global isotopomer approach, no exchange fluxes in reversible reactions can be calculated: one major disadvantage of this approach. METAFoR, net flux calculation and automatic preprocessing of the GC-MS raw data are implemented in the MATLAB-based software FiatFlux [5], which provides a user friendly interface and interactive workflow for the analysis of gas chromatography-mass spectroscopy (GC-MS) detected ^13^C pattern of proteinogenic amino acids. To date, it is preconfigured for the analysis of labeling data of experiments using glucose that is ^13^C-labeled at the C1 position (1-^13^C glucose), and uniformly labeled U-^13^C glucose. The inference of analytical equations describing the ratios of converging fluxes is elaborate and requires expert knowledge of the operative metabolic network of the organism of interest. Recent developments, however, now enable the automated calculation of flux ratios [20] and will greatly facilitate the extension of the METAFoR approach to new metabolic models and carbon sources.

All available software solutions require intense user input and interaction, which are limiting the analysis workflow. Standardization and automation to increase data throughput is needed to transform ^13^C MFA into a high-throughput technology. Here, we show the automation of ^13^C MFA using the software FiatFlux [5] as an example. Accordingly, we give a more detailed introduction to ^13^C-based MFA with the FiatFlux software in the following.

### 1.2. ^13^C-based MFA Using FiatFlux

In brief, the experimental procedure of a ^13^C-labeling experiment is as follows: Biomass is sampled from a steady-state culture of the microorganism growing on specifically labeled glucose. After pretreatment of the biomass to cleave the proteins into the individual amino acids, the sample is analyzed by GC-MS. Every chromatographically separated analyte (*i.e.*, amino acid) eluting from the GC column is analyzed according to its mass in the mass detector. There, the amino acids are ionized via electron impact whereby they are fragmented. These fragments are subsequently separated by their mass to charge (m/z) ratio (e.g., in a quadrupole mass filter). The fragments are analyzed with a mass resolution of at least one mass unit that allows the discrimination of mass isotopomers, that is, of molecules only differing in the number of heavy isotopes.

**Figure 1:**
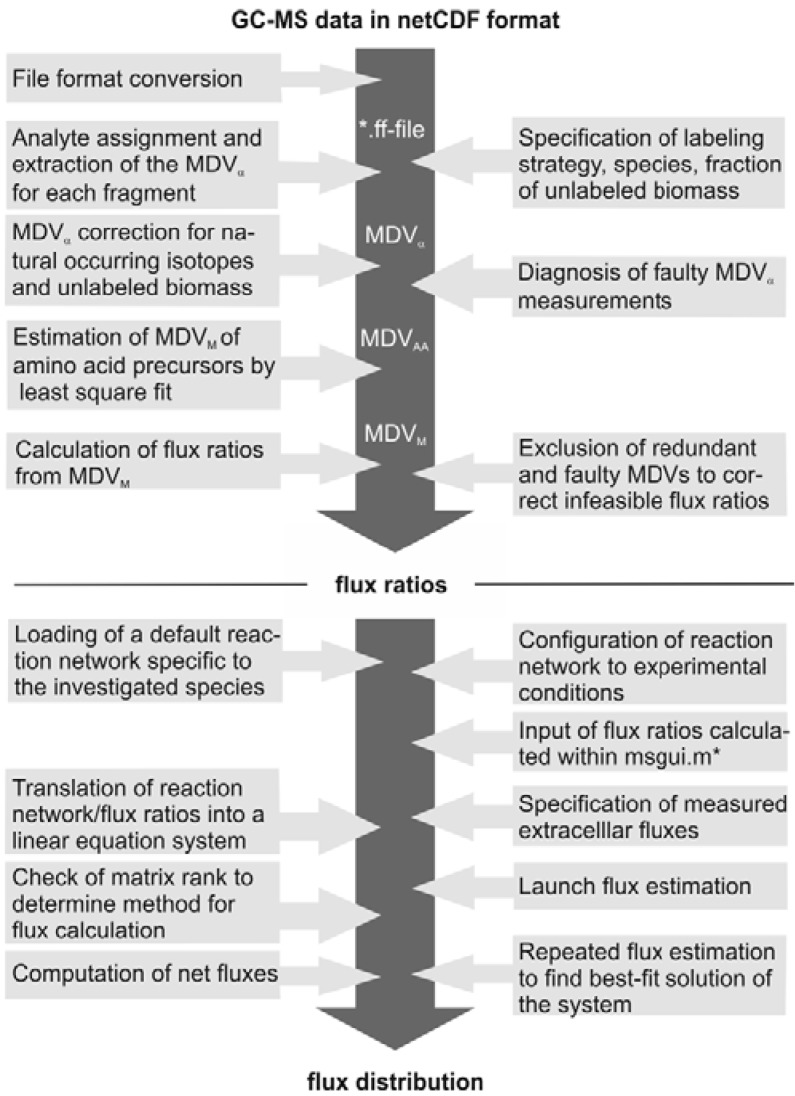
Workflow of ^13^C MFA of FiatFlux [5]. The automatic tasks are listed on the left, the manual user interactions on the right side.

The subsequent FiatFlux analysis workflow can be divided into three major steps of (i) MS data extraction and preprocessing, (ii) metabolic flux ratio analysis and (iii) calculation of the intracellular flux distribution (Figure 1) and is outlined in the following. For an in-depth description of the calculations the reader is referred to [21] and [19]. From the raw mass spectral data, provided in the netCDF format (CDF: common data format), the software automatically detects the amino acid fragments and extracts for each fragment a mass distribution vector (MDV), which lists the fractional abundance of the different mass isotopomers. To determine the mass isotope distribution of the carbon skeleton exclusively originating from the ^13^C-labeled carbon of the carbon source glucose, the MDV are corrected for the natural isotope abundances of oxygen, nitrogen, hydrogen, silicon, sulfur and carbon and the dilution of the isotope signals due to unlabeled biomass introduced with the inoculum. After this data preprocessing steps, metabolic flux ratios are determined. Based on the known atom transitions occurring during the amino acid biosynthesis from central carbon metabolites, the MDVs of these amino acid precursors (MDV_M_) are inferred from the amino acid labeling patterns (MDV_AA_). This is accomplished within FiatFlux by a least square fitting procedure. Metabolic flux ratios are then estimated from these MDVs *via* probabilistic species specific equations.

Net fluxes in the central carbon metabolism network are computed by MFA. Under steady state conditions, the mass balances of the metabolites of a stoichiometric model of an appropriate reaction network form a linear equation system, which is made mathematically accessible by transformation to matrix notation. The system is further constrained by experimentally determined reaction rates and the calculated flux ratios, which are translated into linear equations. Generally, this constrained equation system is fully or even overly determined and a flux distribution is computed by solving the system by a least square optimization. Fluxes are given with confidence intervals estimated from the error of the extracellular fluxes and the flux ratios.

## 2. Results and Discussion

In order to obtain a standardized analysis process and to increase the amount of data sets that can be analyzed in a certain amount of time, the interactive flux analysis procedure has to be automated.

FiatFlux [5], for which we exemplify the automation procedure, is a MATLAB-based software, freely available for academic purposes. The developed automated version of this software has been made remotely accessible by integrating it as services in the Bio-jETI framework [22,23], which has also been used to build the actual analysis workflows. It is based on the jETI electronic tool integration platform [24] and the jABC workflow modeling framework [25]. The name Bio-jETI refers to the distributed nature of many bioinformatics applications, which profit from the easy provisioning and integration of remote services via the jETI technology. We call this process-based ^13^C-flux analysis software, which in its current version allows the user to work with the FiatFlux functionality in a highly flexible and automated manner, Flux-P. In order to make the FiatFlux functionality available for workflow integration, we first developed a “headless" variant of the software, which provides programmatic access to its functions. Afterwards, it was a straightforward process to integrate the required pieces of FiatFlux functionality into the Bio-jETI platform and to use the new services for the definition of the data processing workflows. The development of FiatFlux-Headless, the realization of Flux-P and its evaluation are the content of the three parts of this section.

### 2.1. FiatFlux-Headless

In order to prepare FiatFlux for use from within a workflow environment, we developed a “headless" version of the software, which comprised three major aspects:

(1) *Enabling programmatic access*: Being developed as interactive software, the code has not been designed for invocation from the outside. FiatFlux-Headless makes the functionality of the FiatFlux code base accessible for external invocation (without using the graphical user interfaces (GUIs)).
(2) *Emulation of user interaction*: As the abstract ^13^C flux analysis process depicted in Figure 1 shows, the computations require several user interactions via the GUIs. In FiatFlux-Headless the user interactions that are required at specific points of the original software are emulated by purpose-built methods.
(3) *Visualization support*: FiatFlux comprises functionality for exporting the flux analysis results in different textual formats. As graphical presentations of metabolic flux distributions inside network diagrams are more accessible, FiatFlux-Headless furthermore exports the results in a format that can later be used in network visualization software.

Details on the development of FiatFlux-Headless are given in the following sections.

### 2.2. Enabling Programmatic Access

FiatFlux consists of the two main modules ratio and netto containing the GUI for steering the flux ratio and the net flux distribution computation, respectively. Some other modules provide additional GUI components, for instance for the setting of different experimental parameters, for editing the weights of amino acid fragments that are considered in the flux ratio computation, or for writing the analysis results into text files. Basically, inactivation of the GUI in these modules was achieved by rigorously removing all code for graphical components and replacing invocations from the GUI with (parameterized) functions. These changes made it possible to access the basic FiatFlux functions from external program code and to transfer data between the different analysis steps, which are prerequisites for integration into a workflow environment. Another useful functionality that we adopted from FiatFlux is the possibility to upload custom network models instead of using the standard networks provided by the system. The required information has to be provided as a text file following a defined syntax. Also within this file, the user can specify new metabolic flux ratios complementary to those calculated in the ratio module. The values of such additional ratios have to be provided together with the analytical equations in the model file. Furthermore we extended the FiatFlux outputs with some additional information on the analysis procedure: Initially, results could be exported to text files that contain the data in tab-separated format and can thus be directly imported into spreadsheet programs like MS Excel or OpenOffice Calc. In addition, they are now equipped with information about the quality of the solution expressed by the fitting residual of the least square solutions and the sum of the relative flux errors. If no solution is attained, it is stated whether the reaction system is underdetermined or if no solution could be found that satisfied the defined quality criteria.

### 2.3. Emulation of User Interaction

The user interactions required during the analysis process can be categorized into simple input of experimental data or the selection of modeling parameters and more intricate user interactions targeted to the optimization of the calculations. Whereas the data input could simply be replaced in FiatFlux-headless by parameterized functions (see above), for the other steps the expert logic had to be translated into quantifiable criteria. The functions developed to emulate these tasks are packaged into the two modules *ratio_guiemulation* and *netto_guiemulation*, respectively. In more detail, *ratio_guiemulation* contains the following subfunctions, which are applied in the given order:

*adjust_weights*

Exclude those amino acid fragments from the computation that are obsolete for the used labeling strategy and network model.

*remove_faulty_fragments*

If uniformly labeled glucose has been used, the fractional labeling of the analytes, which should reflect the fractional labeling value of the carbon source, is used as an additional quality criterion implemented in the function *remove_faulty_fragments*. Fractional labeling values that differ from the labeling fraction of the growth substrate by more than a user-defined percentage are not considered in the analysis (Default threshold is 15% deviation from the theoretical labeling fraction.)

*decrease_error*

To obtain flux ratios of high quality, faulty MDV_AA_ have to be excluded from the ratio calculation. In FiatFlux, the quality of the MS data is assessed by inspection of the fitting residuals of each MDV_AA_ plotted on the GUI; fragments with a high residual are inactivated by mouse click on the respective bar. This fragment selection process is automated in the function *decrease_error*. Amino acid fragments with fitting residuals exceeding a defined threshold value (default is 10^−3^) are categorized into unique fragments and redundant or equivalent fragments, hence, fragments with identical carbon backbone origin and theoretically identical MDVs. The most prominent amino acid fragments (denoted as m-15, m-57, m-85, m-159, f302), which evolve due to the electron impact in the MS instrument, are used for the analysis. The fragments m-15 and m-57 contain the complete amino acid carbon chain, while m-85 and m-159 are lacking the carbon atom at position one [11]. Each of these pairs forms a group of equivalent fragments. Moreover, the carbon backbone of amino acids originating from a common precursor, for example L-phenylalanine and L-tyrosine, which are synthesized from prephenate, should be identical after the correction of naturally occurring isotopes. Thus the fragments m-15/m-57 and m-85/m-159 of both amino acids ideally have equivalent MDVs. For redundant fragments, it is checked how many equivalent fragments are used for the analysis. The combination of equivalent fragments that results in the lowest fitting residual is determined and the respective amino acid fragments are excluded from or included in the analysis. Unique fragments not fulfilling the error criterion are directly excluded from the analysis.

*check_ratios*

By definition, calculated flux ratios have to be in the interval from 0 to 1, representing the lower and upper bounds of no or 100% flux through a specific biochemical pathway. Flux ratios not fulfilling this criterion pinpoint to additional faulty MDV_AA_. To eliminate these erroneous MDVs the FiatFlux user has to know the dependency between the MDV_AA_, MDV_M_, and the flux ratios to judge which MDVs have to or can be excluded (e.g., fragments with redundant information). In the function *check_ratios*, emulating this task, flux ratios for which infeasible values are computed are treated as follows: The fragments used for the ratio computation with the highest fitting residuals are stepwise excluded from the computation until the ratio fulfills the error criterion or a flux ratio specific minimum number of fragments is reached. MDVs that are compulsory for the METAFoR calculation are rejected from this selection step. Flux ratios that cannot be corrected are disclosed from the flux distribution calculation.

The module *netto_guiemulation* provides the following subfunctions, which are applied in our application in this order:

*check_ratios*

Excludes ratios from the computation that contain invalid values or that have the value 0.0 and an error of 0.0.

*adjust_balances*

Checks, which experimentally determined extracellular reaction rates are provided. Corresponding metabolite mass balances are adjusted accordingly.

*adjust_reactions*

The default reaction network, specific for the particular organism and automatically loaded by FiatFlux, is adjusted to experimental conditions, for example anaerobic or aerobic environment by exclusion or addition of condition-specific reactions and/or revisions of reaction reversibilities.

*repeated_computation*

The intracellular fluxes are computed by a nonlinear least square fit of the sum of the (weighted) square residuals of the constraints from both metabolite balances and flux ratios starting from a randomly generated flux distribution. As nonlinear problems may have several solutions in the form of local minima of the sum of squares, the optimization problem is calculated a certain number of times and the solution with the lowest fitting residual and lowest sum of relative confidence intervals of the calculated fluxes is saved. Default threshold values for the fitting residual and the sum of the relative confidence intervals are 1 and 0.2, respectively.

### 2.4. Visualization Support

As graphical presentation of metabolic flux distributions greatly facilitates their interpretation, we extended the FiatFlux-Headless code functionality for exporting the calculated fluxes in a format that can be used for visualizing the calculated metabolic flux distributions using the software OMIX [26], an editor for drawing and analyzing metabolic reaction networks. More precisely, the reactions rates together with specific reaction identifiers are exported to a .csv file. This format can easily be interpreted by an OVL (OMIX Visualization Language) script in order to equip default network diagrams with markups according to the obtained results. Via OVL scripts it is, for instance, possible to access and change visual properties (color, shape, line width) of network entities or to assign data to them. The customized metabolic flux charts can then be exported by OMIX into different bitmap and vector graphic formats such as .png, .jpg and .svg.

Within the Flux-P project we currently provide ready-to-use OMIX network diagrams for *B. subtilis*, *C. glutamicum*, *E. coli*, *P. putida* and *S. cerevisiae*, along with an OVL script offering two different markup variants:

- Visualization of a single result data set, where the line width of the reaction arrows is adjusted to the specific flux.
- Visualization of multiple result data sets, where the actual values of the reactions rates are assigned to the arrows representing the respective reaction (see Figure 5).

Note that these OVL scripts work solely on the exported flux distributions, that is, they are completely independent from Flux-P and can be used in other application contexts. In the future, the visualization of calculated flux distributions with OMIX shall be integrated more seamlessly into the Flux-P workflow. Depending on the future development of OMIX this will require either the development of a special plugin or simply the definition of an additional jETI service should OMIX become programmatically or remotely accessible.

### 2.5. Flux-P: MFA Workflows with Bio-jETI

For Flux-P, we used the Bio-jETI technology [22] to make FiatFlux-Headless functions available as a collection of platform-independent remote services and to build user-specific MFA workflows. Bio-jETI is a framework for service integration and workflow development in the bioinformatics domain that has been used in a number of different projects (cf., e.g., [27,28,29]) and is continuously evolving as new service libraries and service and software technologies become established. It is based on the jETI tool integration platform [24] and the jABC modeling framework [25].

### 2.6. Integration of Flux Analysis Services

The jETI technology can be used to make file-based command-line or Java applications remotely available. Therefore the tools have to be registered at a jETI Service Provider Site (SPS), which involves the definition of the commands that are used by the server for the tool invocations, including the parameters and data that have to be passed to the tools. According to the service configurations, XML-based descriptions are generated that contain the information needed by client applications to call the services correctly. At runtime, the jETI SPS receives calls and data from the client, which it forwards to the corresponding registered tool, collects the result, and sends it back to the client. For providing pieces of FiatFlux functionality that are directly accessible by the SPS and that are adequate for workflow modeling, we applied a set of purpose-built scripts to handle (aggregated) function calls and the required data transfers. More precisely, each FiatFlux function of interest (available as a single MATLAB function or as specific sequence of MATLAB functions) is encapsulated by a MATLAB script, which can be executed by MATLAB in headless mode. This invocation of MATLAB is again wrapped as a service into a shell script that can then be called by the jETI SPS. It turned out that a coarse-grained service library, which provides predefined variants of the major analysis steps, rather than exposing computational details of the analysis steps to the workflow level, is advantageous. Thus, we finally provide the following services:

- *MSdataExtraction*: Mass spectrometry (MS) data extraction from .cdf format.
- *METAFoR*: Predefined, complete metabolic flux ratio analysis. Performs the user emulation steps described above.
- *netFlux*: Predefined, complete net flux distribution analysis. Performs the user emulation steps described above.
- *netFlux_CustomModel*: Predefined, complete ^13^C flux analysis based on a user-defined network model. Performs the user emulation steps described above.
- *netFlux_JointRatios*: Predefined, complete ^13^C flux analysis that uses several results from complimentary datasets as input. Performs the user emulation steps described above. Combining data from experiments with different isotopomer mixtures is valuable as it increases the resolution of network fluxes.

The jETI SPS is able to generate clients for the registered services, particularly in the form of jABC workflow building blocks, which take care of exchanging the necessary data with the surrounding workflow and manage the communication with the jETI SPS. The different FiatFlux services can thus be combined with various other services, allowing the user to work with FiatFlux in a highly flexible and automated manner. In the following we give a short introduction to workflow modeling with jABC, before we describe three of the many possible Flux-P workflows that we realized using jABC as a jETI client.

### 2.7. Workflows for ^13^C-data Analysis

**Figure 2:**
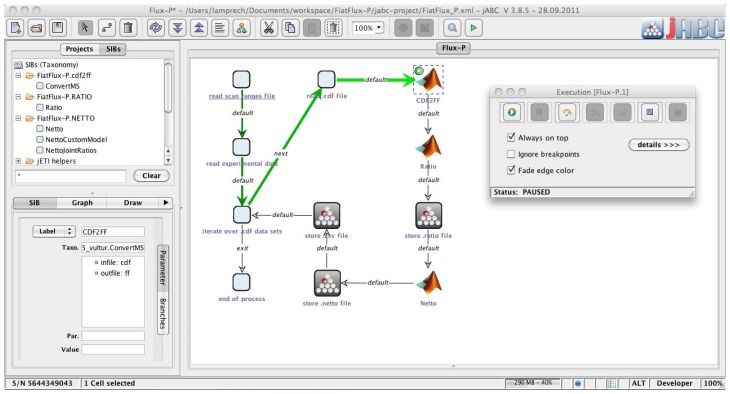
Bio-jETI GUI. The jABC framework, which provides the graphical user interface for Bio-jETI, supports the orchestration of processes from heterogeneous services. Workflow models are constructed graphically by placing process building blocks from a library (top left) on a canvas (center) and connecting them by labeled branches to define the flow of control. The models are directly executable by an inbuilt interpreter component (right).

The jABC framework (Figure 2), which provides the graphical user interface for Bio-jETI, supports the orchestration of processes from heterogeneous services. Workflow models, called Service Logic Graphs (SLGs) are constructed graphically by placing process building blocks, called Service Independent Building Blocks (SIBs), on a canvas and connecting them by labeled branches according to the flow of control.

The SIBs are representatives of accessible functions, at the same time containing the code that calls the corresponding services. The SLGs are thus directly executable by an interpreter component and can be transformed into stand-alone applications or deployed as new services. The jETI SPS can generate SIBs for the registered tools, thus we directly obtain appropriate blocks for the FiatFlux services. The jABC comes with a SIB library providing common control flow and data management functionality. Together, they form a SIB collection for building FiatFlux workflows.

**Figure 3:**
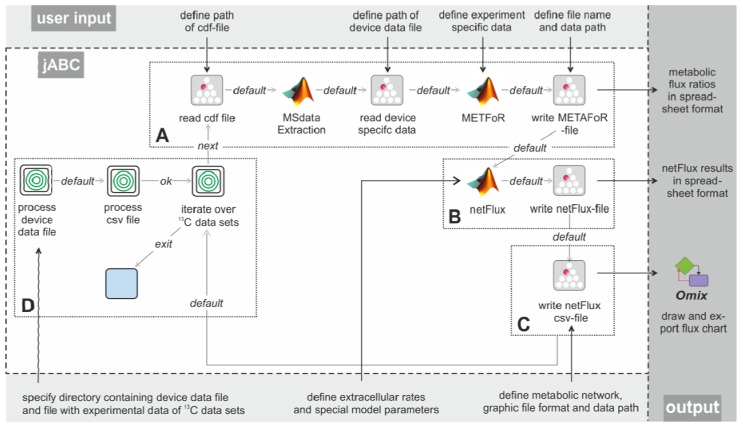
Example workflows for customized ^13^C-based MFA: box A shows a basic workflow for the calculation of metabolic flux ratios of one single dataset. This workflow can easily be extended to fit the user's needs, e.g. to enable net flux calculations (B), visualization of the flux distribution (C) or the batch processing of several datasets (D).

In the following, we describe Flux-P workflows that demonstrate the flexible and easy assembly of Bio-jETI and FiatFlux functions to custom SLGs. These workflow models have been extensively tested for operational reliability and consistency with results from manual analysis. Figure 3 shows the essential parts of the different workflows. The most basic analysis process is realized by the workflow shown in box A, which is restricted to the calculation of metabolic flux ratios: A .cdf file containing the MS data is read by the jETI plugin and sent to the server that runs the MATLAB scripts. First, MS data are extracted from the netCDF file. After the device-specific data of the GC-MS has been read, the METAFoR analysis is performed. The results of the analysis are available as a text file, which is stored to the local file system at the end of the workflow. All required file paths and further information about the experiment are provided via the respective SIB parameters. If physiological data are available, a ^13^C-based MFA via *netFlux* can be performed using the results from the METAFoR analysis. In terms of Bio-jETI processes, simply two SIBs are added to the workflow of Figure 3 that call *netFlux* and store the respective result to the local file system (see box B in Figure 3). By extension of the workflow with box C the net fluxes are saved into a .csv-file. As described earlier, this file can be passed to an OVL script that assigns the reaction rates to the corresponding reaction arrows of a predefined, blank network diagram. Finally, the adopted metabolic network can be converted into a user specified graphic format.

Our actual goal was to realize a workflow that allows batch processing of numerous .cdf data sets. The repeated execution of the FiatFlux computation steps defined in the workflow parts A-C of Figure 3 can be realized without further programming, since the standard library of SIBs that is provided with the jABC software contains a number of functions for often recurring tasks, for example for file management and processing of data collections.

In FiatFlux, all experimental data have to be entered manually by the user at different steps of the analysis procedure and at different parts in the GUI. For Flux-P, we defined a simple table structure that provides the experimental parameters for numerous data sets in a single file. Each line of the table represents one data set and contains a number of defined entries that specify all data required for the analysis. The table has to be stored in a comma-separated format (.csv file). This format can be exported from all common spreadsheet programs, thus researchers can continue to document their experiments within MS Excel, OpenOffice Calc or other. Extension of the workflow in Figure 3 (boxes A-C) with box D enables the processing of several data sets: The user has to specify the working directory, the MS specific data file and the .csv file. The latter is read and split into its lines using a regular expression. Each line (containing the information for one data set) is split into its separate entries (again via a regular expression), which are used as parameters for the Flux-P functions in the current iteration. All these actions are called by the SIB 'process csv file'. As user input is only required once at the beginning, this workflow is able to process very large sets of input data autonomously, speeding up the analysis procedure significantly.

**Figure 4:**
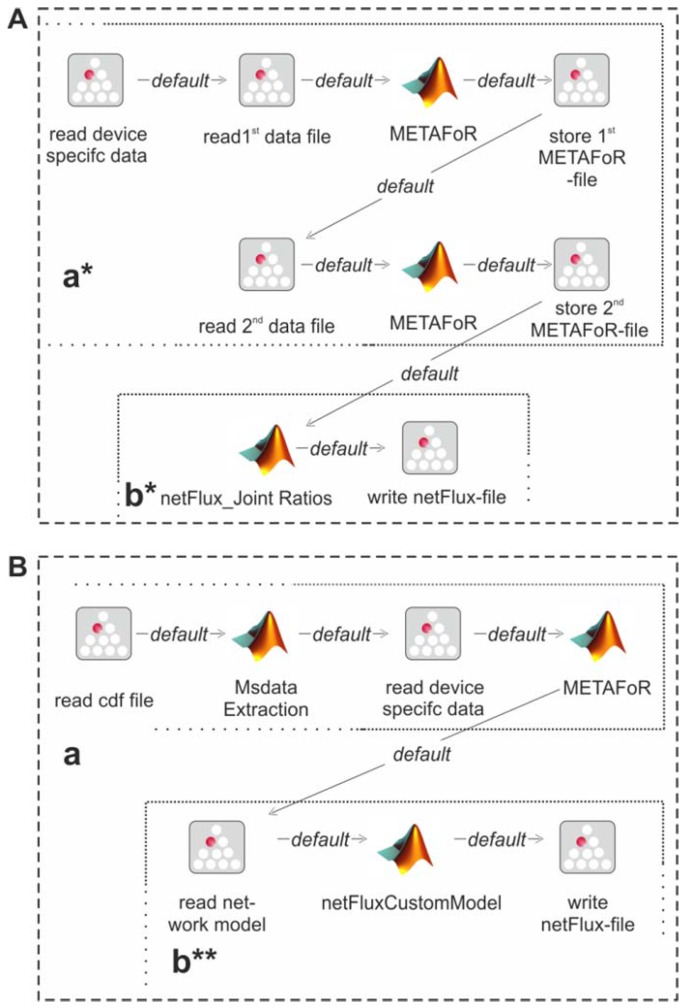
Alternative Flux-P workflows enabling the combined analysis of complementary ^13^C data sets (A) and the use of custom network models (B).

The modular structure of FiatFux [5] allows the calculation of flux distributions using flux ratios of complementary ^13^C labeling experiments. Such a combined analysis is shown in Figure 4A: Metabolic flux ratios of two ^13^C data files are calculated and used together for the subsequent netFlux analysis. Figure 4B shows another workflow variant. Here, instead of using one of the preconfigured networks, a custom metabolic network is uploaded by the user and processed via a special SIB, which translates the content of the text file into the Flux-P model structure. The subsequent analysis is identical to the process described above. Further custom process models are conceivable and can be defined with the same ease as in the illustrated examples. Of course, these workflows variants can also be run in a batch-processing manner as depicted in Figure 3D.

### 2.8. Evaluation of Flux-P

To assess the performance of Flux-P and the reliability of the calculated flux ratios and fluxes, the tool was tested with ^13^C data from labeling experiments with *E. coli*, *B. subtilis* (data of both organisms were taken from [5]), *S. cerevisiae* [30], *Hansenula polymorpha* and two *Pseudomonas* strains. On average, the metabolic flux ratio analysis with Flux-P runs about three to five times faster than the manual analysis. The same holds for the calculation of the flux distribution in *netFlux*. Hence, a complete ^13^C-based MFA (including file upload to the server) performed with Flux-P requires about 4 min instead of 12 to 20 min needed for a manual analysis. As metabolic flux experiments do not only produce a single data set that has to be analyzed, but often 20, 50 or even 150 data sets, this means that the time spent for the data analyses for an experiment is now only about 1:20 h, 3:20 h, or 10 h instead of up to 6:40 h, 16:40 h, or 50 h, respectively. Furthermore the manual analysis requires the full attention of an (experienced) human user, hence it is expensive in the sense that it can easily consume a whole man-week of work. In contrast, the automatic analysis workflows run autonomously in the background, possibly overnight, so that the researcher can focus on other tasks in the meantime.

**Figure 5:**
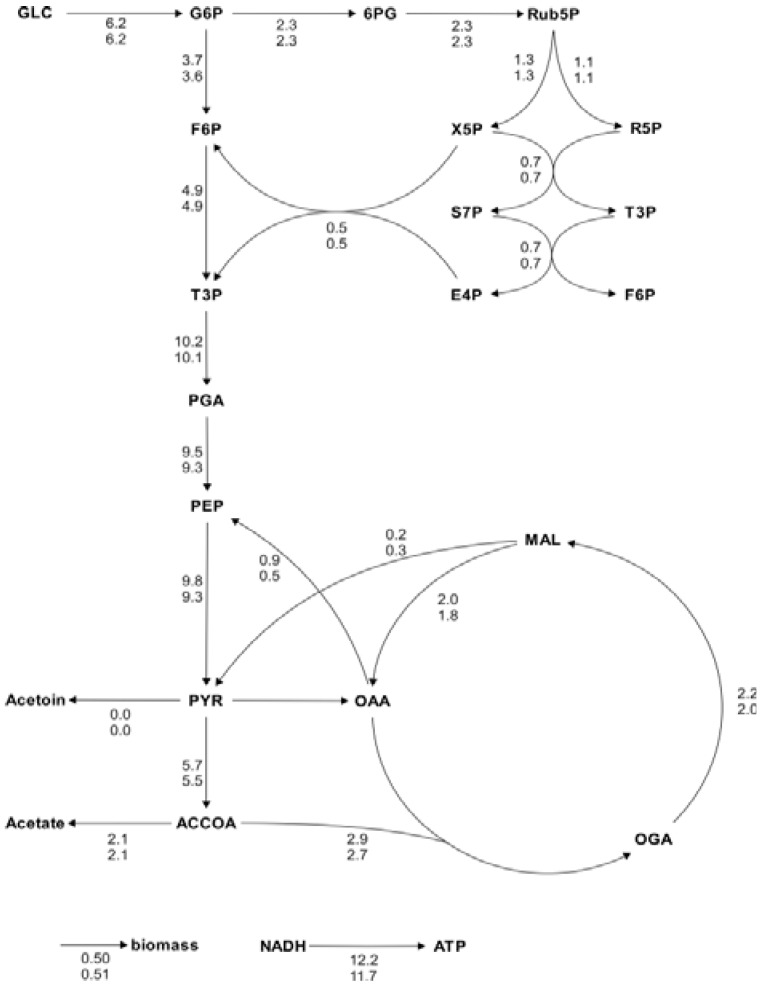
Metabolic flux charts of the *B. subtilis* central carbon metabolism. The flux chart presents data from two flux distributions with the reactions rates plotted next to the reaction arrows. The flux values are given in mmol g_CDW_^-1^ h^-1^ and are calculated with Flux-P (top) and FiatFlux (bottom), respectively. Raw data taken from [5].

**Figure 6:**
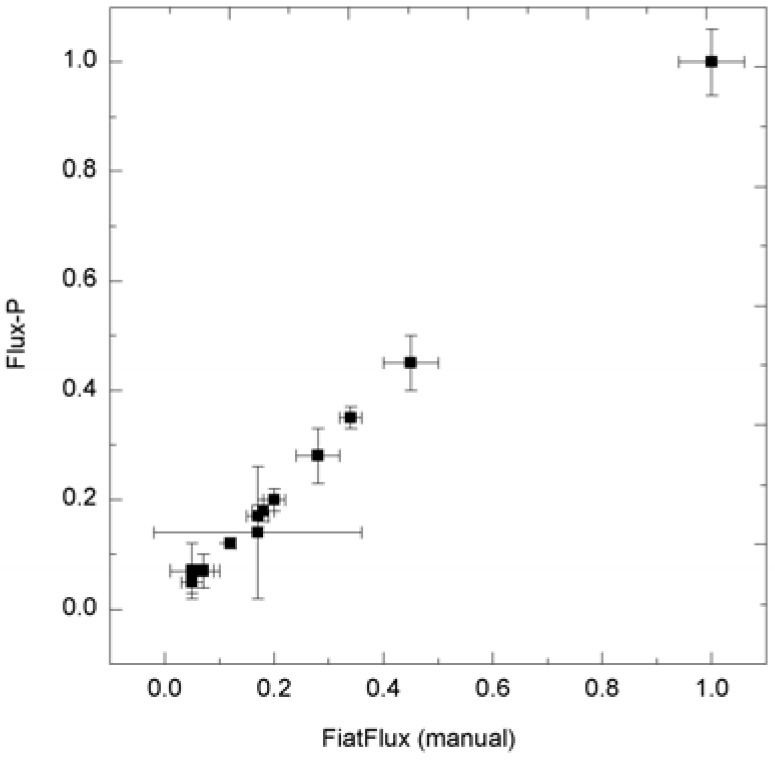
Consistency of metabolic flux ratio analysis calculated with Flux-P. *H. polymorpha* metabolic flux ratios (unpublished data) calculated manually with FiatFlux and with Flux-P show high congruency.

For analysis procedures that do not involve human interaction, it is easy to see that the automation of the in silico experiment using workflow technology increases the speed of the analyses without influencing the results at all. However, workflow realizations of usually interactive analysis processes do not necessarily impact the quality of the results: it is often possible to identify quantifiable criteria in the human expert’s analysis behavior, and apply these for at least heuristic user interaction emulation. The quality of the calculated metabolic flux ratios and intracellular fluxes was investigated by a systematic comparison with the results of the manual analysis. In general, calculated ratios and reaction rates, automatically and manually calculated, coincided quite well. As an example a comparison of automatically and manually calculated flux distributions and metabolic flux ratios are shown in Figure 5 and Figure 6. For the estimation of the *E. coli* and *B. subtilis* flux distributions, data from 1-^13^C and U-^13^C-labeling experiments were available and combined for the analysis (using the workflow shown in Figure 4A). These comprehensive datasets resulted in flux distributions with very high congruency (linear correlation coefficients above 0.99). Moreover, we checked if the data analysis workflow is consistent by repeating the analysis of several datasets 20 times. In all calculations both metabolic flux ratios and fluxes were almost identical with only minor differences in the metabolic flux ratios that did not impact the net flux distribution.

In both examples, minor differences arose due to difference in amino acid fragment selection for the computation and slightly different mass distribution vectors resulting from the automatic data extraction procedure. 

As these examples show, autonomous ^13^C flux analysis—as any automation—entails the risk that raw data of insufficient quality are processed. Therefore, the implementation of routines checking the quality of the original data, e.g. checking for detector overload and data of signals of insufficient intensity are crucial. In Flux-P, MDVs are removed from the analysis, if they cause improper flux ratios assuming a faulty MDV value of this particular fragment. However, equally possible is the use of incomplete or erroneous metabolic networks used for the flux ratio calculation. In order to prevent potentially wrong MDV exclusions and disclosing faulty networks, routines that check alternative network models have to be implemented. In summary, the automated analysis of ^13^C labeling data with Flux-P allows not only a fast pre-screening or initial analysis of large amounts of data but the fully automated calculation of high quality metabolic flux ratios and intracellular fluxes. Observed differences from manually calculated flux distributions can be attributed to shortcomings of the analyzed data to unambiguously resolve all metabolic fluxes rather than to errors in the automated calculation.

## 3. Conclusions

Existing software for ^13^C-based metabolic flux analysis—such as FiatFlux, OpenFLUX or 13CFLUX—supports experts in the complex analysis of intracellular fluxes, but requires several steps that have to be carried out manually, hence restricting their use for data interpretation to rather small numbers of experiments. Flux-P makes it possible to automatically process ^13^C-based MFA of single as well as numerous input data sets. The interactive steps that are essential in the underlying software (FiatFlux in the current prototypical implementation) are replaced by specific scripts that emulate the user interaction, owing to the observation that the user acts, to a considerable extent, according to quantifiable criteria. In addition to the significant acceleration of the analysis process, Flux-P achieves a consistent analysis workflow and applies the same set of parameters to each data set, directly producing comparable results.

We showed that it is easy to integrate software as services via the jETI technology as soon as it can be operated in headless mode. The functions of the software are then available as platform-independent services and can be used for agile workflow definition within Bio-jETI. 

Encouraged by the good results that we have obtained with the prototypic implementation described in this paper, we are going to follow the approach further. Next to the implementation of data quality and model validity checks, discussed in section 2.8, we envisage the implementation of the analytic framework presented by Rantanen *et al.* [20] enabling the automatic derivation of metabolic flux ratio equation for any given metabolic network and any ^13^C labeled carbon source that will extend the current FiatFlux functions. Following the here applied approach, we will integrate alternative flux analysis software into our workflow framework, allowing automated isotopomer balancing. 

As data and results from Flux-P can be flexibly combined with other services, for instance database queries or custom visualizations, extended analyses become possible that exceed the original MFA workflow. Flux-P is unique in supporting flexible changes of the analysis workflows at the user level, which allows researchers to easily adapt their workflows to the changing needs of different analysis setups.

Note that a software system that realizes a MFA workflow based on ^13^C-FLUX2 has recently been described by [31]. The system applies an ActiveBPEL-based process management framework for the implementation of one fixed, comprehensive workflow that integrates ^13^C-FLUX2, the visualization software OMIX and additional, mostly interactive, functionality. 

## Availability

Flux-P is available for academic, non-commercial use and will be provided by the corresponding authors on request. Note that a FiatFlux license is required.

Flux-P consists of a server running the underlying analysis software and requiring a particular setup, and the client-side workflows that can be run on any machine. On the server side, the software requires a Unix-based operating system (Linux, Unix, Solaris, Mac OS X), a recent Java Runtime Environment (JRE), MATLAB R2011a or later (including the MATLAB Optimization and NetCDF Toolboxes), a recent Java Runtime Environment (JRE) and the Flux-P jETI server. On the client side, the software requires a recent JRE and the Java Application Building Center (jABC), Bio-jETI release, version 3.8.1 or later (available from [23]). The Flux-P workflows are platform-independent and have been tested on Windows 7, Ubuntu Linux and Mac OS X.

## Supplementary Files

Supplementary File 1 Supplementary File (PDF, 4337 KB)
