# Supplementary material for: Flux-P: Automating Metabolic Flux Analysis
Source: Metabolites. 2012 Nov 12;2(4):872–90. doi: 10.3390/metabo2040872 (PMC3901227; doi:10.3390/metabo2040872)
Supplement: Supplementary File 1 — Supplementary File (PDF, 4337 KB) [file metabolites-02-00872-s001.pdf]

Flux-P

Quick User Guide to Automated ^{13}C-Based Metabolic Flux Analysis with Flux-P

Birgitta E. Ebert, Anna-Lena Lamprecht *, Bernhard Steffen and L. M. Blank *

* Author to whom correspondence should be addressed; E-Mail: lars.blank@rwth-aachen.de (L.M.B.); Tel.: +49-241-80-26600; Fax: 49-241-80-22180.

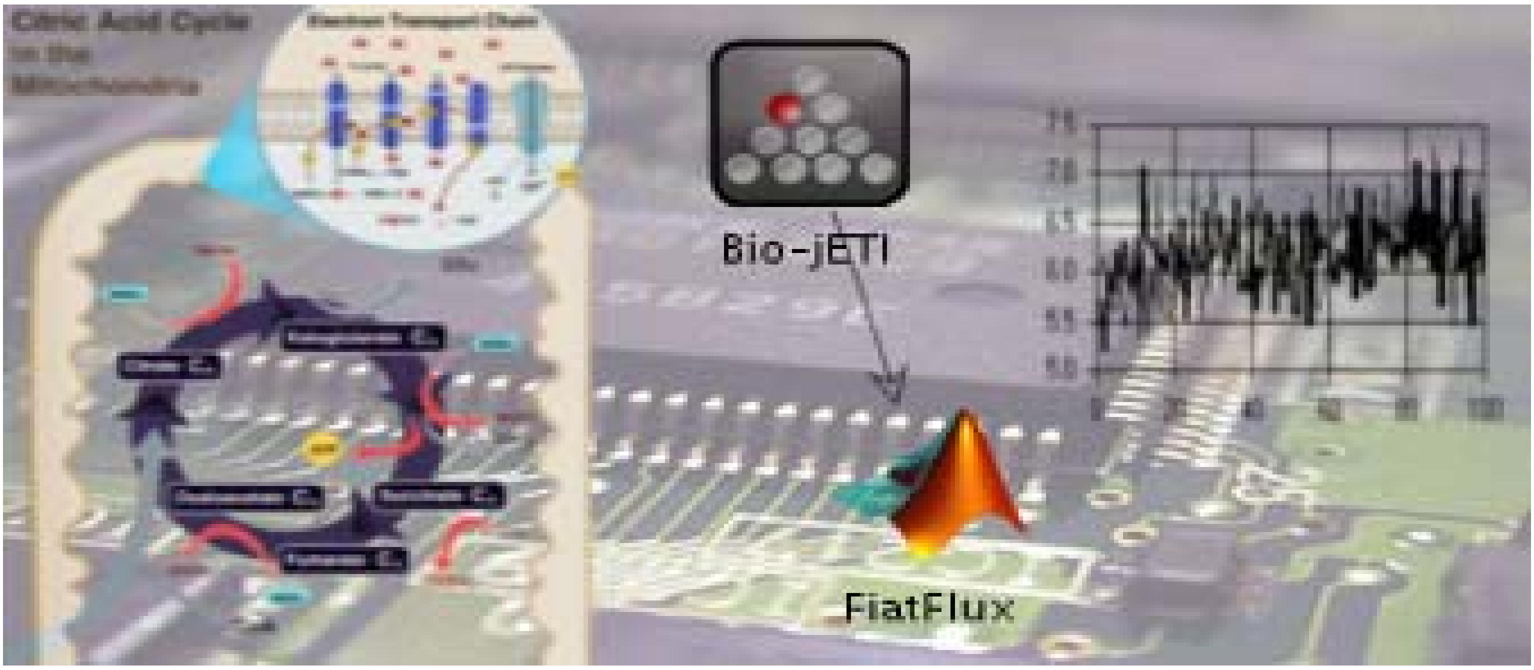

This is a quick user guide for automated ^{13}C-based metabolic flux analysis using Flux-P. Release 1.0 provides services for automating FiatFlux [1], software for metabolic flux analysis of data from ^{13}C-glucose experiments.

The ^{13}C data analysis workflow of FiatFlux has been subdivided into single process steps and the required user interaction has been translated into computational functions. These programmatically accessible units have then been made available as platform-independent remote services using the (Bio-) jETI technology [2] and can be easily assembled to user-specific metabolic flux analysis workflows via the jABC modeling framework [3].

In form of a small tutorial, this user guide gives instructions for the use of Flux-P workflow models, explaining the steps of four basic Flux-P workflows.

Thank you for using Flux-P.

Requirements

- Recent Java Runtime Environment (JRE)
- Java Application Building Center (jABC), Bio-jETI release, version 3.8.1 or later, available from http://ls5-www.cs.tu-dortmund.de/projects/biojeti/download.php
- Flux-P server and Flux-P.zip archive containing the client-side jABC/Bio-jETI project files. Available on request from Anna-Lena Lamprecht (lamprecht@cs.uni-potsdam.de) or Lars M. Blank (lars.blank@rwth-aachen.de). Matlab and FiatFlux licences required.

Installation

jABC/Bio-jETI installation

Install the jABC/Bio-jETI software, simply by launching the installer and following the directions of the installation wizard.

Import Flux-P project

Unzip the Flux-P.zip archive and add the resulting directory as a new project via 'Project: new project' (see Figure 1). The project and the included example workflows appear in the project browser (see left upper panel of the GUI shown in Figure 3) and can be used without any further adjustments.

In some cases, the Flux-P specific SIBs are not automatically added to the SIB path. In this case add the SIB folder contained in the Flux-P folder manually (via Project > Add SIB-Path; see Figure 1).

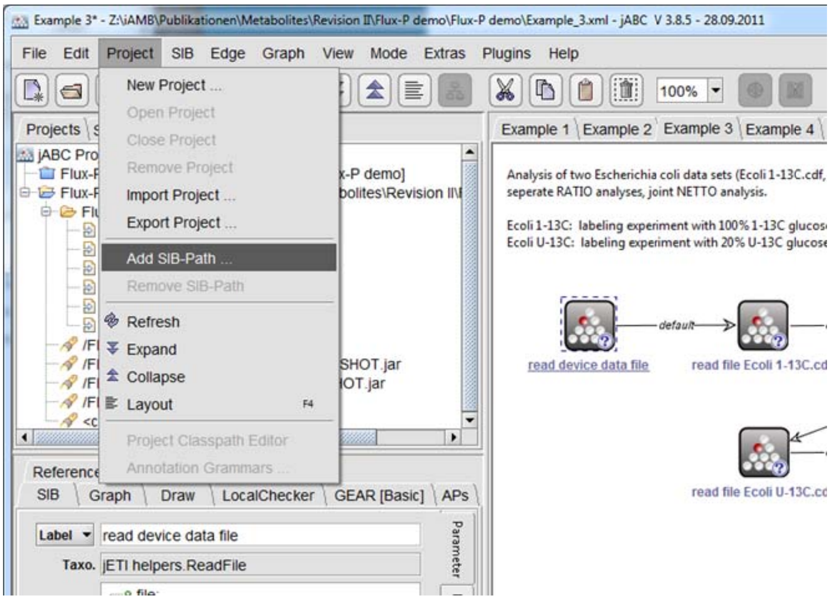

Figure 1 Manual addition of Flux-P specific SIBs.

Automated ^{13}C-based metabolic flux analysis with Flux-P

In this section, we introduce Flux-P applications by describing the necessary steps of sample ^{13}C flux analysis workflows. The introduction details the workflows of single METAFoR and/or netFlux analyses, the use of custom models for the netFlux analysis and the combination of complementary ^{13}C datasets for the estimation of a common flux distribution and the batch processing of several datasets. Finally, we describe the procedure for the visualization of flux distributions.

Introduction into general jABC functionality

Please refer to the jABC manual for a general introduction to the software (available at http://jabc.cs.tu-dortmund.de/manual/index.php/Category:Manual).

The screenshot in Figure 3 shows the graphical user interface of jABC. After import of the Flux-P project the included models appear in the project browser (1). These models are activated by double mouse click after which the respective workflows appear on the canvas (2). The start SIB is underlined by a continuous line. If alternative workflows are contained in the model, the start SIB can be selected and deselected by choosing 'Start SIB' from the context menu (right mouse click on the SIB).

A model is executed by pressing the Run button (A) on the toolbar (see Figure 2). Alternatively, the tracer (B) can be used with which the workflow is processed stepwise.

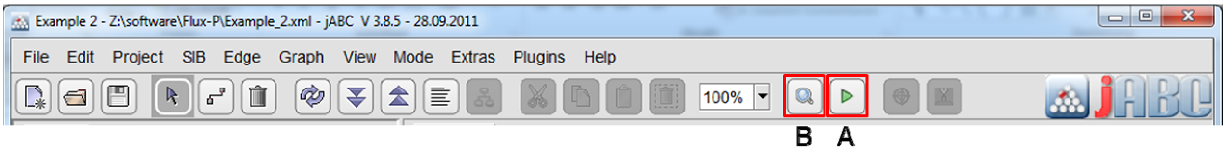

Figure 2 Execution of models via Run button (A) or tracer (B).

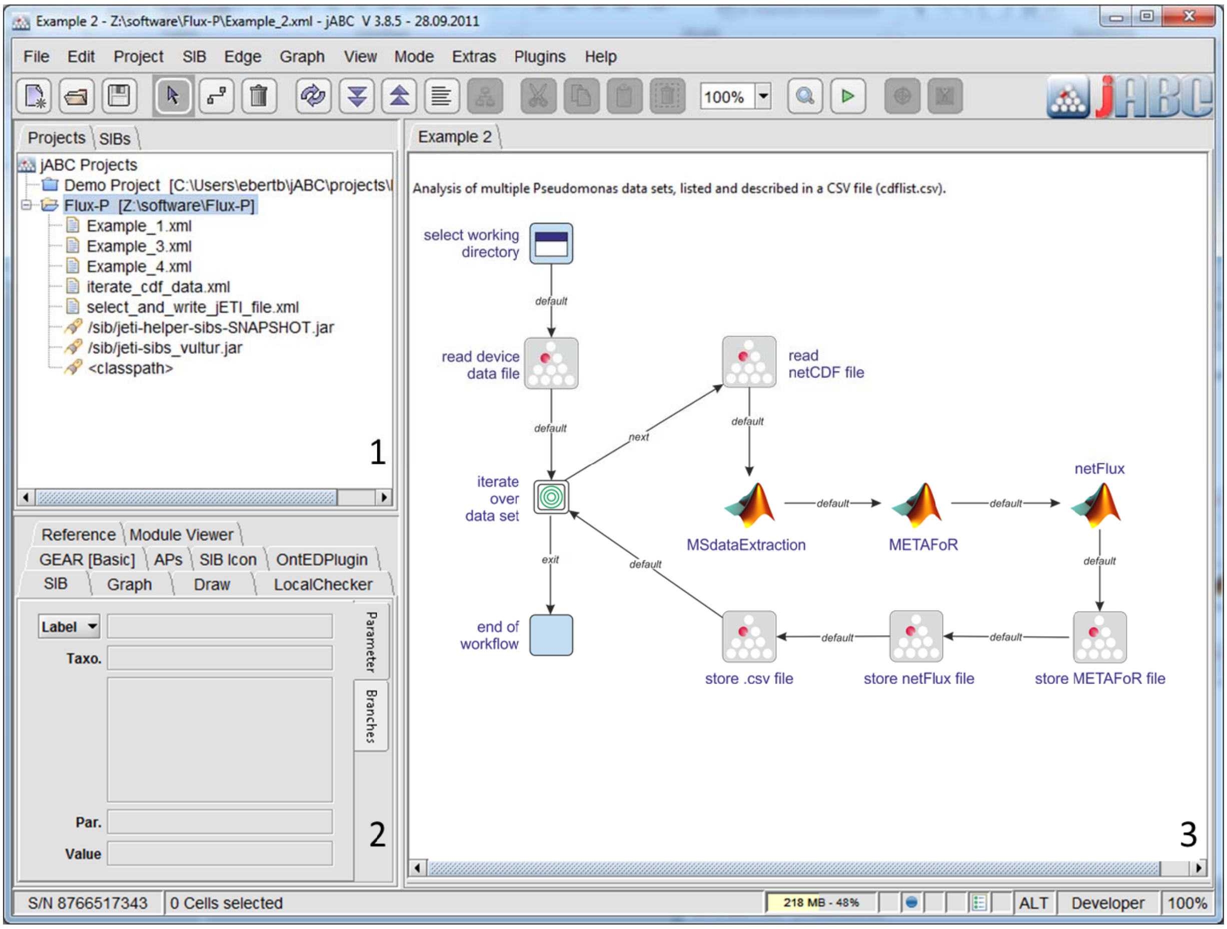

Figure 3 The jABC user interface with its main components project and SIB Browser (1), inspector pane (2) and graph canvas (3).

Standard METAFoR and netFlux analysis

The Flux-P model for the analysis of a single ^{13}C data set consists of 7 SIBs shown in workflow 1 shown in Fiugure 4.

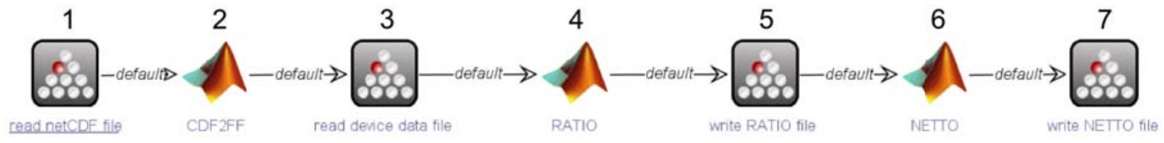

Figure 4 Workflow 1: METAFoR and netFlux analysis of single ^{13}C dataset

The single steps and the required input of the user are detailed in the following:

Step 1: Upload of the local netCDF to the server

Activate SIB 'read netCDF' by mouse click. In the inspector pane, particular properties of the SIB appear. Here the user has to specify the data path of the netCDF (see Figure 5).

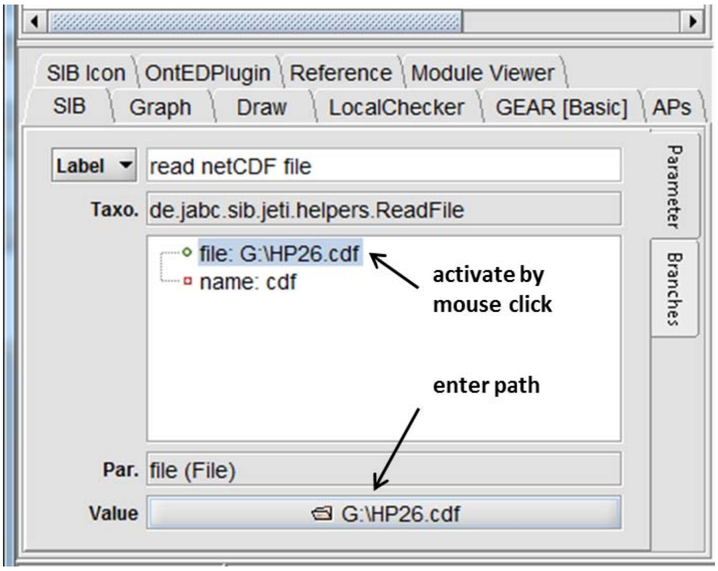

Figure 5 User input for netCDF file selection.

Step 2: Data extraction from the netCDF file

Step 3: Upload of GC-MS specific data

Analogous to step 1, the path of the device data file is specified. This file contains the scan ranges of the analyzed amino acids, which are specific to the device and method used.

A properly formatted file (saved as .txt file) is shown in Figure 6

.

```
ALA 1 50
GLY 20 100
ASP 850 950
GLU 1010 1110
THR 630 730
TYR 1570 1670
SER 600 700
PHE 750 850
MET 550 650
HIS 1400 1600
VAL 100 200
LEU 150 250
ILE 210 310
PRO 250 350
LYS 1200 1300
```

Figure 6 Format of device data file.

Step 4: METAFoR analysis

As can be seen from the screenshot shown in Figure 7, a bunch of information has to be given for a proper METAFoR analysis. This data are entered into the inspector pane of the 'METAFoR' SIB.

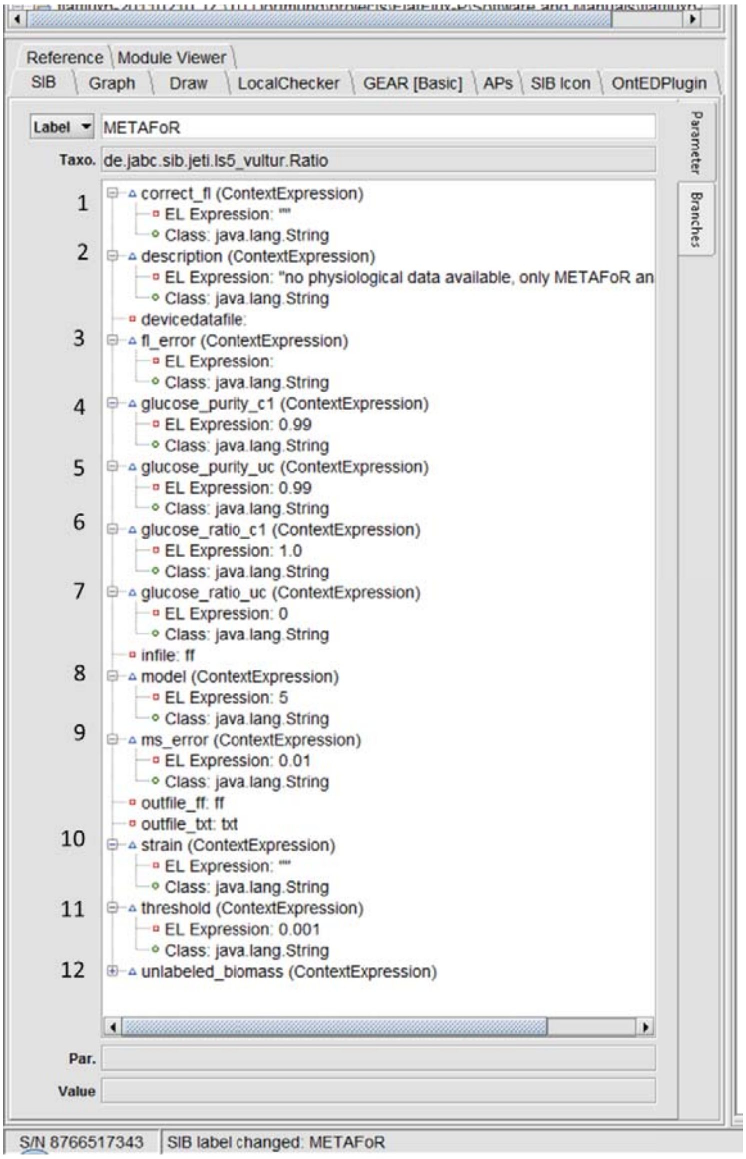

1. Amino acid supplements in the medium result in diluted fractional labeling (fl). An appropriate correction factor can be entered here.
2. Description of the experiment (optional)
3. Absolute error allowed for the fl of amino acid fragments. Frgaments with higher error are excluded from the analysis.
4. Purity of the 1-^{13}C glucose, default is 99%
5. Purity of the U-^{13}C glucose, default is 99%.
6. Molar ratio of 1-^{13}C glucose in the medium.
7. Molar ratio of U-^{13}C glucose in the medium
8. Model to be used for the calculation of metabolic flux ratios (1 *Bacillus*, 2 *Escherichia*, 3 *Sacchoromyces cerevisiae* 4 *Zymomonas*, 5 *Pseudomonas*, 6 *Rhodobacter*, 7 *Corynebacterium*, 8 *Thiobacillus*)
9. Measurement error of the mass spectrometer, default is 0.01.
10. Name of the strain (optional)
11. Amino acid fragments with a fitting residual above the specified threshold are excluded from the analysis. Default value is 0.001.
12. Mass fraction of unlabeled biomass

Figure 7 User input for METAFoR analysis

Step 5: METAFoR results are stored as text file on the local system.

Analogous to step 1, the data path and name of the METAFoR file has to be entered.

Step 6: NETTO analysis

The following user input is required:

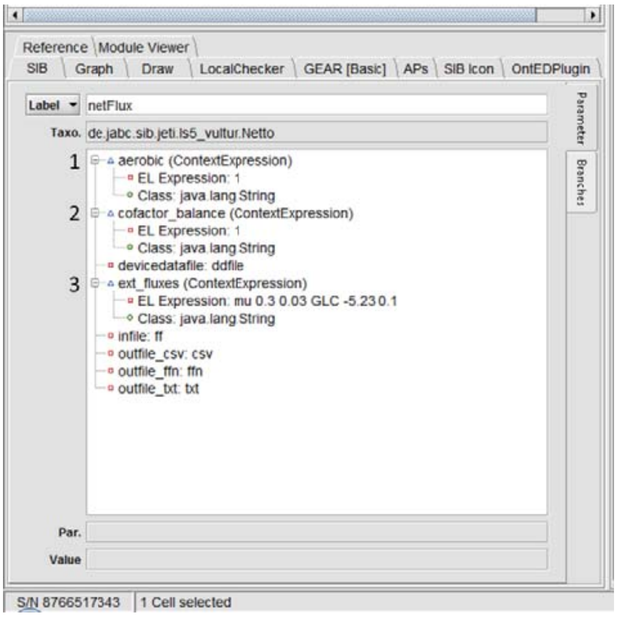

1. For experiments with *E. coli* two different model variants are selected if the experiment was performed under aerobic or anaerobic conditions, respectively.
2. The user can specify, if either the ATP, NADH mass balance or both should be excluded from the analysis. (0 ATP balance disclosed, 1 NADH balance disclosed, 2 ATP and NADH balance disclosed)
3. Extracellular fluxes used as additional constraint for the linear equation system are entered here. The syntax of the string is: {metabolite name} {rate in mmol gCDWh-1} {standard deviation of rate}. 'mu' is used to enter the growth rate.

Figure 8 User input for netFlux analysis

Step 7: netFlux results are stored on the local system.

Data path and name are entered analogous to step 5.

Upload of custom models

The Flux-P project provides metabolic networks for eight different species. However, custom models can easily be uploaded using the workflow 2 in Figure 9.

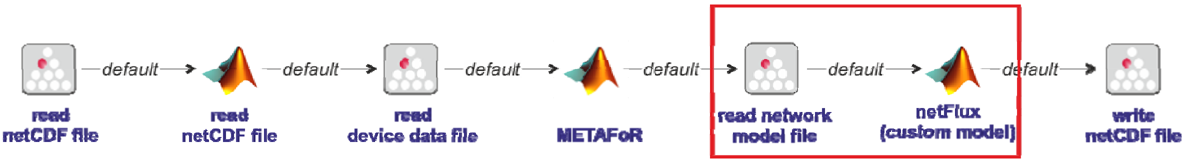

Figure 9 Workflow 2: ^{13}C data analysis using a custom metabolic network.

The new network model has to be specified according to the syntax defined by Zamboni [1] and saved as .txt-file.

For further guidance for the model setup, we refer the user to the FiatFlux manual.

netFlux analysis using METAFoRS of complementary ^{13}C datasets

The resolution of the intracellular fluxes is dependent on the design of the ^{13}C tracer experiment. While use of 1-^{13}C glucose allows the unambiguous calculation of the glucose-6-phosphate partitioning into Emden-Meyerhof-Parnas, pentose-phosphate and Entner-Doudoroff pathway, for the calculation of the fluxes into the TCA cycle mixtures of uniformly labeled glucose and naturally glucose are better suited. According to Fischer et al. [4], the best resolution is attained, if duplicate experiments with the two complementary labeling strategies are applied. The automated analysis of the resulting ^{13}C datasets is depicted in the workflow below.

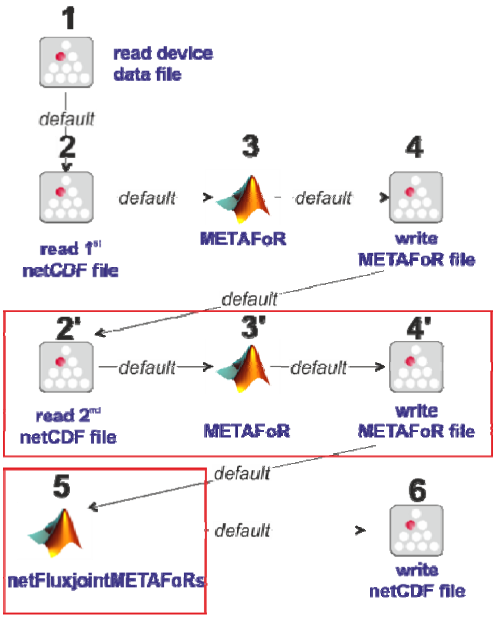

Figure 10 Workflow 3: netFlux analysis using METAFoRs of complementary ^{13}C datasets.

Data input is identical to the workflow 1 described above. In comparison to workflow 1, the steps 2-4 are duplicated and the SIB 'netFlux' is replaced with the SIB 'netFluxJointMETAFoRs' that can process two METAFoR files.

Batch processing

To enable batch processing of several datasets, the data which had to be entered manually in the inspector pane in the illustrated examples, is collected in one single file, which is read and interpreted by the SIBs 'process .csv' file' and 'process data sets (see workflow 4 in Figure 11).

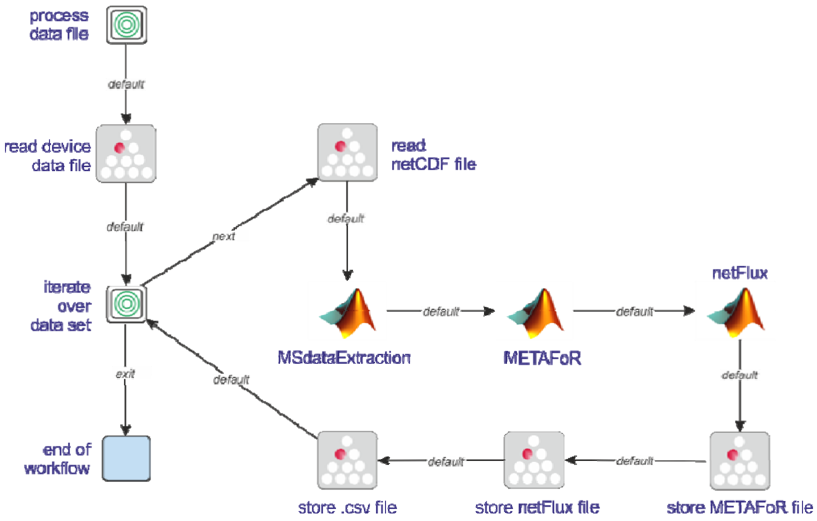

Figure 11 Workflow 4: Batch processing of several ^{13}C datasets.

The structure of the .csv file containing the gathered information is depicted in Figure 12 and explained in Table 1. The 1st line is the header line specifying the different entries. Some of these header names are fixed whereas, e.g. the extracellular fluxes are dependent on the experiment and are defined by the user.

cdffile	description	strain	model	fraction_1_13C	purity_1_13C	fraction_U_13C	purity_U_13C	fraction_unlabeled_biomass	GCMS_error	growth_rate
1	continuous cultivation	*P. putida* KT2440	5	0	0.99	0.2	0.99	0.01	0.01	0.5
2	continuous cultivation	*P. putida* KT2440	5	0	0.99	0.2	0.99	0.01	0.01	0.5
3	continuous cultivation	*P. putida* KT2440	5	0	0.99	0.2	0.99	0.01	0.01	0.1
4	continuous cultivation	*P. putida* KT2440	5	0	0.99	0.2	0.99	0.01	0.01	0.1
5	continuous cultivation	*P. putida* KT2440	5	0	0.99	0.2	0.99	0.01	0.01	0.2
6	continuous cultivation	*P. putida* KT2440	5	0	0.99	0.2	0.99	0.01	0.01	0.2
7	continuous cultivation	*P. putida* KT2440	5	0	0.99	0.2	0.99	0.01	0.01	0.3
8	continuous cultivation	*P. putida* KT2440	5	0	0.99	0.2	0.99	0.01	0.01	0.3
9	continuous cultivation	*P. putida* KT2440	5	0	0.99	0.2	0.99	0.01	0.01	0.4
10	continuous cultivation	*P. putida* KT2440	5	0	0.99	0.2	0.99	0.01	0.01	0.4
11	continuous cultivation	*P. putida* KT2440	5	0	0.99	0.2	0.99	0.01	0.01	0.05
12	continuous cultivation	*P. putida* KT2440	5	0	0.99	0.2	0.99	0.01	0.01	0.05

Figure 12 Structure of data file for batch processing of ^{13}C data.

Table 1 Specification of entries of the data file for ^{13}C data batch processing

Header[a]	Entry	Remark
cdffile	name of netCDF file	Enter only the name without .cdf ending. Only word characters, i.e. [a-zA-Z_0-9]+) are allowed
description	short description of experiment	optional[b]
strain	name of the investigated strain	optional[b]
model	specify model no for use of the metabolic flux ratio analysis	1 *Bacillus*<br>2 *Escherichia*<br>3 *S. cerevisiae*<br>4 *Zymomonas*<br>5 *Pseudomonas*<br>6 *Rhodobacter*<br>7 *Corynebacterium*<br>8 *Thiobacillus*
fraction_1_13C	molar fraction of 1-^{13}C glucose	value has to be in the interval [0;1]
purity_1_13C	purity of 1-^{13}C glucose	default values is 0.99
fraction_U_13C	molar fraction of U-^{13}C glucose	value has to be in the interval [0;1]
purity_of U_13C	purity of U-^{13}C glucose	default values is 0.99
growth_rate[c]	specific growth rate [h^{-1}]	
growth_rate_error[c]	standard deviation of growth rate	

glucose_uptake_error[c]	glucose uptake rate [mmol g_{CDW} h^{-1}]	
cofactor_balance	Select, which cofactor mass balance is excluded from the analysis	0 disclose ATP balance<br>1 disclose NADH balance<br>2 disclose ATP and NADH balance

[a] Only word characters, i.e. [a-zA-Z_0-9]+) are allowed; do not use space.
[b] Type ' ' to submit no information.
c Column headers of extracellular rates can be named freely. Here only growth rate and glucose rate are listed, additional rates are specified accordingly.

The netFlux SIB entry 'ext Fluxes' has to be adapted to allow proper assignment of the extracellular fluxes (see Figure 13). The syntax of the string is exemplified here for the sample csv-file shown in Figure 12:

mu ${cdfdata.growth_rate} ${cdfdata.growth_rate_error} GLC ${cdfdata.glucose_uptake_rate} ${cdfdata.glucose_uptake_rate_error}

'mu' denotes the growth rate. For substrate uptake and product formation the identifier of the respective metabolic network is used.

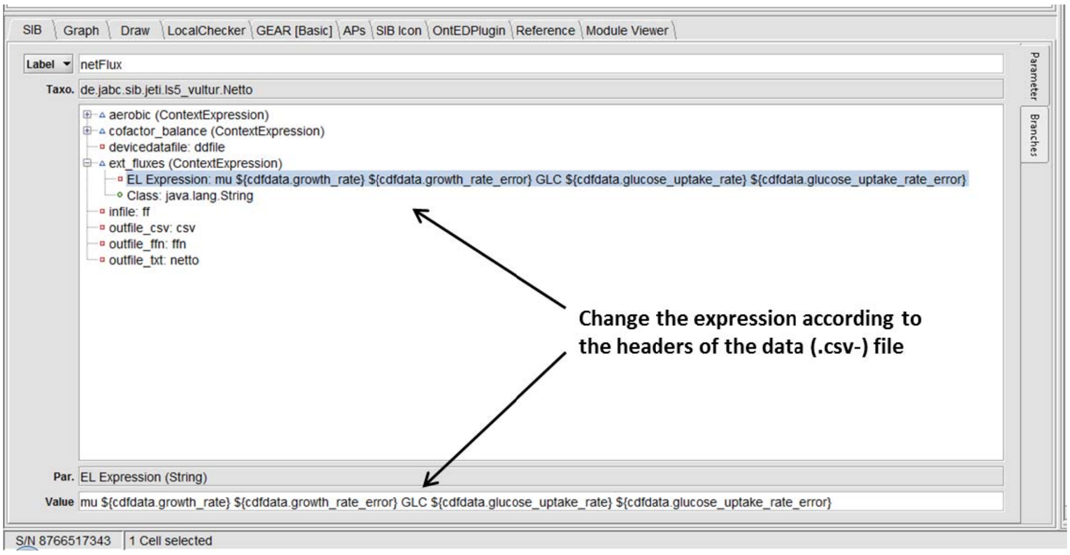

Figure 13 The expression of the ext_fluxes in the netFlux SIB has to be adapted according to the identifiers of the extracellular rates in the csv-file.

Visualization of flux distributions with OMIX

OMIX [5] is an editor for drawing and analyzing metabolic reaction networks, available from http://www.13cflux.net/omix/. It can be used for visualization of the metabolic flux distributions calculated with Flux-P.

In addition to the classical, FiatFlux-style textual results, the different netFlux SIBs can export the calculated reaction rates together with specific reaction identifiers into .csv files. These can then be interpreted by OVL (OMIX Visualization Language) scripts, which can, for instance, equip default network diagrams with markups according to the obtained results. Via OVL scripts it is, for instance, possible to access and change visual properties (color, shape, line width) of network entities or to assign

data to them. The customized metabolic flux charts can then be exported by OMIX into different bitmap and vector graphic formats such as .png, .jpg and .svg.

In the scope of the Flux-P project we have prepared ready-to-use OMIX network diagrams for *B. subtilis*, *C. glutamicum*, *E. coli*, *P. putida* and yeast, which are readily equipped with an OVL script that offers two different markup variants:

1. Visualization of a single result data set, where the line width of the reaction arrows is adjusted to the specific flux.

2. Visualization of multiple result data sets, where the actual values of the reactions rates are assigned to the arrows representing the respective reaction.

As Figures 14 and 15 show, OMIX displays two buttons for invoking the functions provided by the OVL script, in this case for starting the visualization of a single or of multiple results. When choosing to visualize a single result, the user will be asked to define the .csv file containing the result. When choosing to visualize multiple results, the user will be prompted for the number of results to be visualized first, and then asked to provide the .csv files. The Figures also give an example of the effect of the described markups.

Note that these OVL scripts work solely on the exported flux distributions, that is, they are completely independent from Flux-P and can deliberately be used in other application contexts.

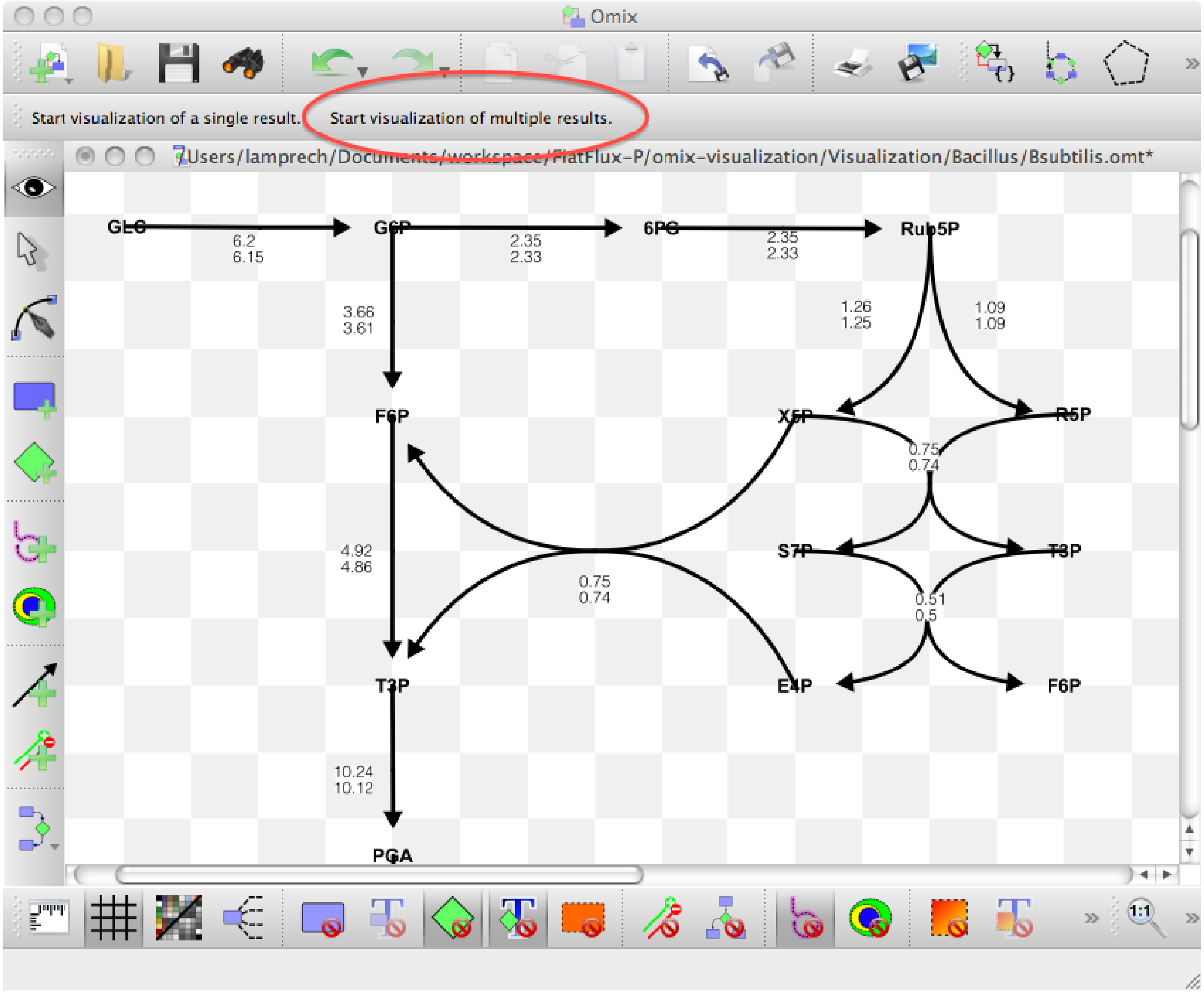

Figure 14 Visualization of a single result data set in OMIX.

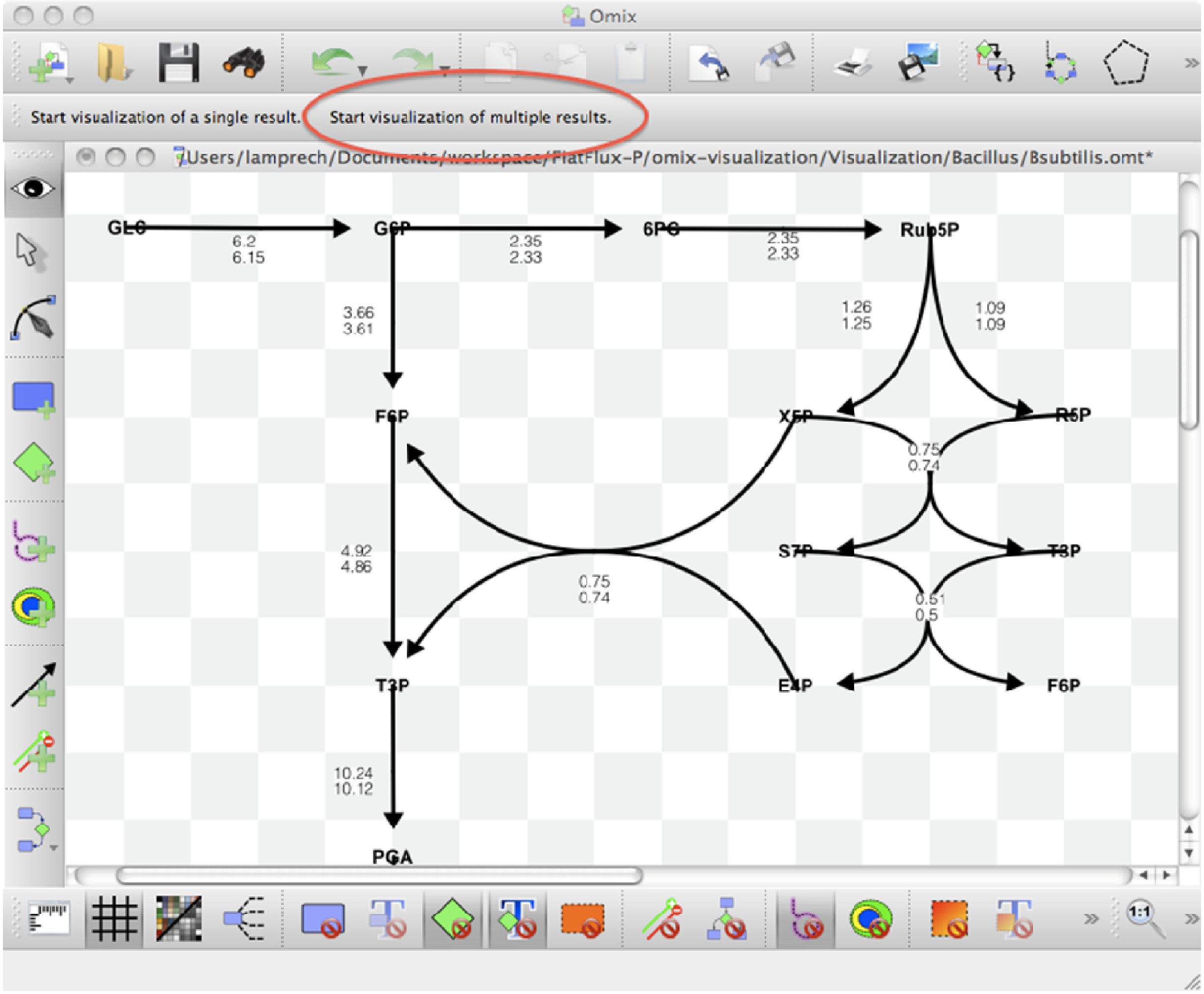

Figure 15 Visualization of multiple result data sets in OMIX.

Additional information to FiatFlux-SIBs

Table 2 FiatFlux services provided by the jETI SPS.

Service	Description
MSdataExtraction	Extract mass spectrometry data from the netCDF file
METAFoR	Predefined, complete METAFoR analysis.
netFlux	Predefined, complete netFlux analysis.
netFlux_CustomModel	Predefined, complete netFlux analysis with customized network model.
netFlux_JointMETAFoRs	Predefined, complete netFlux analysis using two METAFoR results as input.

References

1. Zamboni, N.; Fischer, E.; Sauer, U. FiatFlux--a software for metabolic flux analysis from ^{13}C-glucose experiments. *BMC Bioinformatics* **2005**, *6*, 209.
2. Margaria, T.; Kubczak, C.; Steffen, B: Bio-jETI: a service integration, design, and provisioning platform for orchestrated bioinformatics processes. *BMC Bioinformatics* **2008**, 9 (Suppl 4), S12.

3. Steffen, B.; Margaria, T.; Nagel, R.; Jörges, S.; Kubczak, C. Model-Driven Development with the jABC. In: *Hardware and Software, Verification and Testing*. Springer: Berlin/Heidelberg, Germany, 2007; pp. 92-108.
4. Fischer, E.; Zamboni, N.; Sauer, U. High-throughput metabolic flux analysis based on gas chromatography-mass spectrometry derived 13C constraints. *Anal. Biochem.* **2004**, *325*, 308-316.
5. Droste, P.; Noack, S.; Noh, K.; Wiechert, W. Customizable Visualization of Multi-omics Data in the Context of Biochemical Networks. *Visualisation* **2009**, 21-25.
